# Nitrogen Excretion by Dairy Cows Grazing Plantain (*Plantago lanceolata*) Based Pastures during the Lactating Season

**DOI:** 10.3390/ani12040469

**Published:** 2022-02-14

**Authors:** Soledad Navarrete, María Rodriguez, David Horne, James Hanly, Mike Hedley, Peter Kemp

**Affiliations:** School of Agriculture and Environment, Massey University, Palmerston North 4442, New Zealand; jimeroge@gmail.com (M.R.); d.j.horne@massey.ac.nz (D.H.); j.a.hanly@massey.ac.nz (J.H.); m.hedley@massey.ac.nz (M.H.); p.kemp@massey.ac.nz (P.K.)

**Keywords:** plantain-based pastures, dairy cows, nitrogen excretion, nitrogen leaching, grazing systems

## Abstract

**Simple Summary:**

Plantain (*Plantago lanceolata*) has emerged as a forage with the ability to reduce nitrogen (N) losses, in particular N leaching, from grazing dairy systems. For farmers to confidently incorporate plantain into their farms, research needs to demonstrate that these environmental benefits sustain similar production and farm profit than traditional ryegrass (*Lolium perenne*)–white clover (*Trifolium repens*, wc)-based pasture. The effect of changing the cows’ diet to plantain-based pastures was evaluated over two lactation seasons in comparison to ryegrass–wc pastures. Cows grazing plantain-based pastures produced the same milk solids production, but a urine with a lower N concentration when compared to ryegrass–wc pastures. Plantain reduced the urinary N loads from individual urine patches via higher urine volume and reduced the total N loading onto pastures via a lower urinary N excretion to mitigate the N leaching losses from grazed pastures. Plantain pastures could be employed by farmers to reduce the nitrate leaching from dairy farms.

**Abstract:**

The use of plantain pasture in dairy systems can potentially reduce nitrogen (N) leaching losses via the lower N concentration in the urine (UNc) of cows. Reducing the urinary N load while cows graze pastures can reduce the risk of N leaching losses from urine patches. Research needs to demonstrate that these environmental benefits are not at the expense of milk production and farm profit. Three groups of 20 cows grazed in the following three pasture treatments: (i) plantain, (ii) plantain–clover mix (plantain, red [*Trifolium pratense*] and white clover), or (iii) ryegrass-white clover (wc) pastures, from spring to autumn for two years. Each year, pasture intake, diet quality, milk production and animal N (milk and urine) excretion were evaluated in spring, summer, and autumn. The cows grazing the plantain and plantain–clover mix pastures produced similar milk solids as cows grazing ryegrass–wc pasture but reduced their UNc during summer and autumn, when compared to those grazing the plantain–clover mix and ryegrass–wc pastures. Plantain reduced urinary N loads onto pastures by a greater number of urine patches with lower urinary N loading rates. The results demonstrate that plantain pastures do not diminish milk solids production from cows, and the lower UNc from summer to autumn could reduce N being lost to the environment.

## 1. Introduction

New Zealand (NZ) pastoral farmers are committed to adopt farming practices that improve the quality of surface and ground waters draining their land. To achieve this environmental target for freshwater quality, dairy systems need to reduce nitrogen (N) loss, principally via nitrate (NO_3_^−^) leaching from grazed pastures [1]. In dairy systems, based on grazing ryegrass (*Lolium perenne*)–white clover (*Trifolium repens*) pastures, the largest contributor to NO_3_^−^ leaching is the surplus of dietary N in the pasture relative to the cow’s requirements, with the surplus N excreted onto pastures via the urine of the cow [2]. Strategies developed to reduce the NO_3_^−^ leaching from pastoral systems involve reducing the urinary N load while cows are at pasture. These strategies include the feeding of low protein concentration supplements or crops with pasture diets [3], or the housing of cows when not actively grazing to minimize the time they are on the pasture [4], but both these strategies either greatly increase the costs of feed supply or of farm infrastructure [5,6].

Dairy farming reliance on pasture-based diets has created the opportunity to evaluate plant species that alter the urinary N excretion from cows in a manner that mitigates N losses from grazing animals. Cow urination produces highly concentrated N patches, with N loading rates that exceed the pasture’s ability for N uptake, leaving a mineral N surplus in the soil that contributes to NO_3_^−^ leaching during periods of soil drainage [6]. Reducing both the concentration of N in urine (loading rate) and/or the total N loading into the soil will lead to a reduced risk of nitrate-N leaching from pastoral systems [5,6]. In the last decade, plantain (*Plantago lanceolata*)-based diets have been demonstrated to decrease urinary N concentration while not affecting milk production, relative to ryegrass-white clover pastures, in short-term studies [7,8,9]. A longer-term farm system experiment over 2 years showed milk production was unaffected by including plantain in the diet, and lower urinary N concentrations were predicted but were not measured [10].

The ability of plantain to reduce the urinary N concentration of lactating cows has been associated with the forage having a diuresis effect and/or reducing the amount of N excreted in urine [11,12]. Plantain herbage increases urine volume of cows, producing a more diluted urine. Modelling research has suggested that reducing the N concentration in urine via greater urine volume could lead to a reduction in N leaching [6]. A higher water-soluble carbohydrate (WSC) and lower soluble protein concentration may also reduce the total urinary N excretion from lactating cows by changing the N partitioning in cows [13]. Plantain contains secondary bioactive compounds (aucubin and acteoside) with antimicrobial properties that are likely to be playing a role in altering N partitioning during ruminal fermentation [14]. Reducing the urinary N concentration (UNc) of cows by grazing pastures containing plantain creates an opportunity to modify pastures and decrease the urinary load of grazing cows in a cost-effective manner. 

Reducing the urinary N concentration of cows by grazing pastures containing plantain could be used by farmers to decrease the urinary load of grazing cows in a cost-effective manner. However, for farmers to confidently change their farms to plantain-based pastures, the effect of plantain on urinary N concentration and milk production for the full lactation season needs to be evaluated. This research explores for first time the effect of grazing plantain-based pastures on decreasing the urinary N loads from lactating cows to mitigate N leaching from pastoral soils. This study evaluated the urinary N losses from lactating cows grazing plantain and plantain clover mix pastures, including white and red clover (*T. pratense*) pastures, in comparison with perennial ryegrass-white clover (wc) pastures over two lactation seasons, and the role of plantain bioactive compounds in influencing cows’ urinary N loading in a dairy system experiment.

## 2. Materials and Methods

### 2.1. Experimental Design and Treatments

A dairy system experiment was conducted at the Massey University Dairy Farm No 4 in Palmerston North, New Zealand (40°23′ S; 175°36′ E) with the approval of the Massey University Animal Ethics Committee (Protocol 16/137). The soil type at the research site is an artificially drained, Tokomaru silt loam, which is classified as an Argillic-fragic Perch-gley Pallic Soil. A detailed description of soil physical properties is provided by [15]. The site is non-irrigated and has been in permanent pasture for more than 90 years.

The experiment evaluated the following three pasture-based diets (treatments): (i) plantain (cv. Tonic; 10 kg/ha), (ii) plantain–clover mix, containing plantain (cv. Tonic; 8 kg/ha), red clover (cv. Tribute; 4 kg/ha) and white clover (cv. Sensation; 4 kg/ha), and (iii) perennial ryegrass (25 kg/ha)-white clover (3 kg/ha) pastures under grazing with lactating cows.

The three pasture treatments were established (1 December 2016) using a complete randomized design (CRD) with five replicate plots (40 × 20 m), and the area surrounding the experimental plots was divided into three outside areas of approximately 1.0 ha each. These three outside areas were sown to each pasture treatment and, in each grazing rotation, were used to adapt the cows’ diet to the pasture treatments for six days before placing the cows (4 cows/plot) in the experimental plot treatments.

### 2.2. Cows and Grazing Management

Sixty multiparous lactating cows (Friesian × Jersey) were selected from the Dairy Farm No 4 herd to be grazed in the pastures throughout two full lactation seasons (i) from September 2017 to May 2018 (2017/2018 season) and (ii) from September 2018 to May 2019 (2018/2019 season). Cows were separated in three groups (*n* = 20 cows/group) balanced for lactation number, days in milk (DIM), milk production, and body weight (BW). Each group of cows grazed one of the three pasture treatments for 8 consecutive days with a grazing interval of approximately 4–5 weeks, in each grazing rotation. The pastures were grazed on 7 occasions in the 2017/2018 season and 8 times in the second (2018/2019) season.

At each grazing rotation, the cows first grazed in the outside area for 6 days (D1–D6) before spending 2 days (D7–D8) in the experimental plots (4 cows/plot). Cows transitioned from the herd diet to the pasture treatments over a 3-day period (D1–D3). During D1 to D3, cows grazed in a ryegrass–wc pasture after the morning (AM) milking and in their pasture treatments after the afternoon (PM) milking. From D4 onwards, the cows grazed after AM and PM milking in their pasture treatments. The pasture diets of the cows were supplemented with the same quantities (5–8 kg DM/cow/day) of supplements in each grazing rotation, which depending upon season included maize silage, dairy pellet, dried distiller grains (DDG) and baleage, peas, and soy hull. Once the 8-day grazing rotation was completed, all the cows were returned to the farm herd until the next grazing rotation.

### 2.3. Pasture Management

During the 2017/2018 season, the three pasture treatments received two applications of 30 kg N/ha as AMMO31 (60:40, ammonium sulphate: urea, 31% N) during spring (8 August and 12 October 2017), and one application of 35 kg N/ha as urea in summer (12 January 2018), totalling 95 kg N/ha/y. In the 2018/2019 season, 30 kg N/ha as urea was applied to the ryegrass-white clover and plantain pasture on 28 August 2018, and on 4 October 2018, 50 kg N/ha was applied only to the plantain plots as urea, totalling 110 kg N/ha/y. The ryegrass-white clover plots were topped with a mower during the 2017/2018 season on 6 November and 12 December 2017, and on 1 October 2018 of the 2018/2019 season, to maintain pasture quality.

### 2.4. Sample Collection and Measurements

#### 2.4.1. Pasture Treatments

Herbage mass (kg DM/ha) pre- and post-grazing at each grazing period and the botanical composition and nutritive value of each pasture treatment were determined for each experimental plot. Before and after each grazing, three herbage samples (quadrats 0.1 m^2^) were randomly taken from each plot by cutting to ground level, with an electric shearing handpiece, all the herbage inside a 0.1 m^2^ quadrat. All herbage samples were individually washed, to remove soil contamination, and oven dried at 70 °C for approximately 48 h to determine DM production. The botanical composition and chemical composition of the pasture were evaluated from all experimental plots during the 2017/2018 and 2018/2019 seasons in the following periods: (i) spring (September 2017 and 2018), (ii) early summer (December 2017 and 2018), (iii) late summer (February 2018 and 2019) and (iv) autumn (March 2018 and May 2019).

For botanical composition, three herbage samples were taken from each plot a day before the grazing by cutting the herbage (quadrats 0.1 m^2^) at ground level using an electric shearing handpiece. Each sample for botanical composition was manually separated into each pasture species (ryegrass, plantain, white clover, red clover, weeds) and dead material, and the components oven-dried individually at 70 °C (approximately 48 h). The proportion of each species in the total dry matter (DM) was then calculated.

One grab sample (200 g fresh weight) of herbage from each plot was taken pre-grazing, oven dried, and ground to pass a 1 mm sieve for chemical composition. Ground samples were analysed individually for N, metabolizable energy (ME), neutral detergent fibre (NDF), WSC, non-structural carbohydrates (NSC) using near-infrared spectrophotometry (NIRS) [16]. The crude protein (CP) percentage was estimated by multiplying %N by 6.25. The aucubin concentration was determined in plantain and plantain–clover mix herbage samples by high-performance liquid chromatography (HPLC) as described by [14].

#### 2.4.2. Lactating Cows

Cows were scheduled to be milked twice daily, at 0700 and 1500 h, during grazing periods, but in the first (2017/2018) lactation season, all cows were on once a day (OAD) milking at 0700 h from December 2017 until the end of the season as a consequence of a lack of rain in late spring. In the second (2018/2019) lactation season, towards the end of lactation, in May 2019, the cows were placed on OAD milking. Milk, urine, faeces, and blood samples were collected from all cows throughout the two lactation seasons (2017/2018 and 2018/2019) during the grazing rotations in the follow periods: (i) spring (September 2017), (ii) early summer (December 2017 and 2018), (iii) late summer (February 2018 and 2019) and (iv) autumn (March 2018 and May 2019).

Milk yield (L/day) from each cow was recorded at each milking with an automated system (DeLaval Alpro^TM^ system, Tumba, Sweden) during the grazing rotation. On day 8 (D8), during the AM and PM milking, milk samples (35 mL) were collected from each cow for analyses. All milk samples collected during the AM and PM milking from each cow were analysed for milk solids (MS) (fat + protein) and urea concentration using spectrometric analyses (FT120 analyser, Foss Electric, Hilleroed, Denmark).

Urine and blood samples from each cow were collected on day 7 (D7) and D8 of the grazing rotation immediately after the AM milking. Spot urine samples were obtained by manual stimulation of the vulva. One set of urine samples was acidified with sulphuric acid (6N H_2_SO_4_) to reduce the urine pH to 3.0–4.0 and the other set was prepared with normal urine (non-acidified) to later analyse for N, urea and creatinine concentrations, respectively. Urine samples collected from each cow, on D7 and D8, were analysed individually for N concentration using a Leco combustion analyser (AOAC, 2000; method 968.06). The creatinine concentration in urine samples was determined calorimetrically by the Jaffe reaction [17].

Blood samples (10 mL) were taken from the coccygeal vein of each cow and centrifugated at 3000× *g* for 15 min at 4 °C. Then, a sample of plasma was transferred into 2 mL micro-tubes and stored at −20 °C until later analysis of urea. The urea concentrations in the urine and plasma were determined using the Urease Kinetic UV assay.

### 2.5. Calculations and Statistical Analysis

The herbage removed (kg DM/ha) by cows at each grazing, calculated from the difference between herbage mass pre grazing and post-grazing in each plot, was summed over time to estimate the total herbage harvested from each pasture treatment by lactating cows during the 2017/2018 and 2018/2019 season. Pasture DM intake (DMI), estimated as Kg DM/day = herbage removed (kg DM/ha) × plot area (ha)/number of cows (*n*) × grazing days in each grazing rotation (*n*), was used to estimate the daily N intake and the proportion of plantain in the total diet of the cows.

Daily urine volume produced by cows was estimated as L urine/day = 21.9 (mg/kg) × BW (kg) × 1/urinary creatinine (mg/kg) as previously described by [18], which was then multiplied by UNc (g/L) to obtain the daily urinary N (UN) excretion (g N/day) from cows. The N in milk (g N/day) and the UN excretion (g N/day) were divided by the daily N intake (g N/day), obtained from cows grazing in the experimental plot, to estimate the N use efficiency (NUE) and the fraction of N partitioned to urine in lactating cows.

Data were analysed using the PROC MIXED procedure of SAS 9.3 using the model for the complete randomised experimental design. Differences between the pasture treatments in the total herbage harvested in the 2017/2018 and 2018/2019 lactation seasons, and the daily N intake, proportion of plantain in the diet, and aucubin concentration, were evaluated within each season (spring, summer, and autumn). The model included the pasture treatment (plantain, plantain–clover, and ryegrass–wc pastures) as fixed effects and the experimental plot as a random effect. Urine, plasma, and milk parameters were analysed using repeated measures (D7 and D8) within each season (spring, summer, autumn) evaluated and included as subject the cows.

Means were compared using the least squares means test and significance was declared at *p* < 0.05.

## 3. Results

### 3.1. Pasture Treatments and MS Production

During the 2017/2018 season, differences in the ME content between the pasture treatments were seen only in early summer 2018 (Table 1). During the 2018/2019 season, the ME content in plantain pastures was lower than in ryegrass–wc during spring and early summer 2018. In late summer 2019, plantain and plantain–clover mix pastures had greater ME content than ryegrass–wc. The ME content was similar in the three pasture treatments in autumn 2019. Both plantain and ryegrass–wc pastures had a similar CP concentration in all seasons evaluated, except in spring 2017. The CP concentration in plantain pastures was lower than ryegrass–wc pastures in spring 2017, while the CP concentration in the plantain–clover mix pastures was greater than plantain and ryegrass–wc pastures in spring, early and late summer of the 2017/2018 season. During the 2018/2019 season, the CP concentration in plantain–clover mix pastures was greater than ryegrass–wc in early summer 2018 and autumn 2019. The NDF and NSC concentrations in plantain and plantain–clover mix pastures were lower and higher than in ryegrass–wc, respectively. However, in early summer 2018, the NDF concentration in plantain–clover mix pasture was the same as in ryegrass–wc, the NSC concentration was lower when compared to plantain and ryegrass–wc pastures.

The herbage mass harvested by lactating cows during the 2017/2018 season was similar for the ryegrass–wc (10.9 ± 0.22 t DM/ha) and plantain (10.1 ± 0.20 t DM/ha) pastures, but 17% more herbage was harvested by cows from the plantain–clover mix pasture (12.7 ± 0.39 t DM/ha) (Table 2).

In the 2018/2019 season, cows grazing in the ryegrass–wc (10.3 ± 0.81 t DM/ha) and plantain–clover mix pastures (10.2 ± 0.91 t DM/ha) harvested 19% more herbage than cows grazing plantain (8.3 ± 0.27 t DM/ha) pastures. The MS production (kg MS/cow) from cows after 8 days grazing in the three pasture treatments is presented in Table 2. There were no differences in the MS production between plantain, plantain–clover mix, and ryegrass–wc pastures in all seasons evaluated during the 2017/2018 and 2018/2019 seasons.

The proportion of plantain in the diet was similar for cows grazing plantain and plantain–clover mix pastures, except in late summer 2018, when the proportion of plantain in the diet of cows grazing in plantain–clover mix pastures was lower than for plantain pastures (Table 2). Aucubin concentration in plantain and plantain–clover mix pastures was similar during spring and early summer, but in late summer and autumn, aucubin concentration was higher in plantain than plantain–clover mix pastures (Table 2).

### 3.2. Nitrogen Use and Excretion 

The N intake by cows was similar when grazing ryegrass–wc and plantain pasture in all seasons evaluated during the 2017/2018 and 2018/2019 lactation seasons (Table 3). Cows grazing in the plantain–clover mix pasture had greater N intake in late summer and autumn during the 2017/2018 season, and in early summer of the 2018/2019 season when compared to those grazing plantain or ryegrass–wc pastures.

The milk N output from cows was similar among the three pasture treatments, except during spring 2017 and late summer 2018 (Table 3). In spring 2017, the N milk was lower in cows grazing plantain compared to those grazing plantain–clover mix and ryegrass–wc pastures. In late summer 2018, the milk N output was lower in cows grazing plantain and ryegrass–wc than plantain–clover mix pastures. Differences in the NUE between the three pasture treatments were found during the 2017/2018 lactation season, but not in the 2018/2019 (Table 3). During the 2017/2018 lactation season, the NUE from cows grazing plantain and plantain–clover mix pasture was greater in spring but lower in late summer, when compared to cows grazing ryegrass–wc pasture. In early summer and autumn, the NUE was lower on plantain–clover mix pasture compared to plantain and ryegrass–wc pastures. 

During the 2017/2018 lactating season, the total N excreted by lactating cows grazing plantain and plantain–clover mix pastures in spring and early summer was the same as those grazing ryegrass–wc pastures (Table 3). In late summer and autumn, the UN excretion from cows grazing plantain and plantain–clover mix pasture was lower than ryegrass–wc. During the 2018/2019 lactation season, the UN excretion was greater for cows grazing plantain and plantain–clover mix pastures than cows grazing ryegrass–wc in early and late summer; however, in autumn the UN excretion was lower for cows grazing plantain and ryegrass–wc pastures than plantain–clover mix pastures. Differences in the N partitioned to urine between plantain and ryegrass–wc pastures were found in the 2017/2018 and 2018/2019 lactation seasons. During the 2017/2018 lactation season, cows grazing plantain pasture partitioned more N to urine compared to those grazing ryegrass–wc and plantain–clover mix pastures in spring; but in summer (early and late) and autumn, the N partitioned to urine was higher in ryegrass–wc, intermediate in plantain, and lowest in plantain–clover mix pasture. In the 2018/2019 lactation season, cows partitioned more N to urine when grazing in plantain and plantain–clover mix pastures (Table 3).

**Figure 1:**
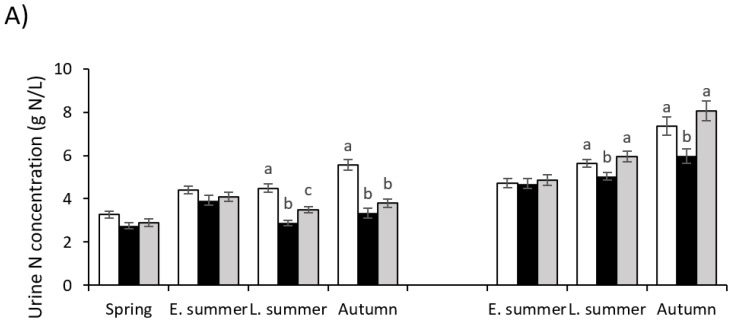

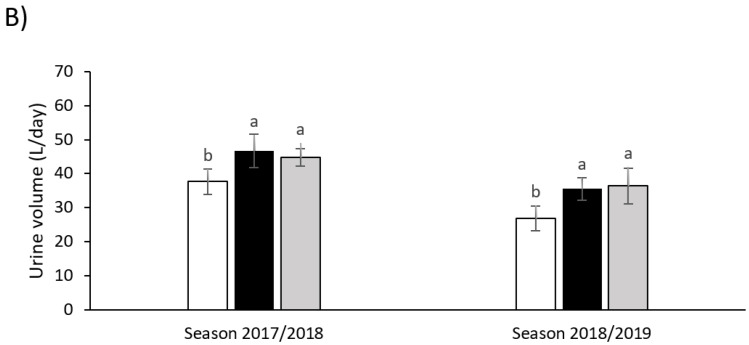
(**A**) Urinary N concentration and (**B**) urine volume from lactating dairy cows grazing plantain (black), plantain–clover (grey), and ryegrass–white clover (white) pastures in spring, summer (early and late), and autumn over two years. a,b,c letters indicate values in each season that are significantly different (*p* < 0.05).

The UNc for cows grazing plantain and plantain–clover mix were the same as for those grazing ryegrass–wc pastures in spring (*p* = 0.07) and in early summer of 2017/2018 (*p* = 0.17) and 2018/2019 (*p* = 0.88) lactation seasons. Both plantain and plantain–clover mix pastures significantly reduced the UNc of cows in late summer and autumn when compared with ryegrass–wc pastures (Figure 1). During the 2017/2018 season, the UNc was, on average, 29 and 36% lower in late summer (*p* ≤ 0.0001) and autumn (*p* ≤ 0.0001), respectively, in cows grazing both plantain and plantain–clover mix pastures than ryegrass–wc pasture. In the second (2018/2019) season, the urine from cows grazing plantain pastures was 13 and 23% lower in late summer (*p* = 0.03) and autumn (*p* = 0.007), respectively, when compared with cows grazing ryegrass–wc or plantain–clover mix pastures. The urine production (L/day), estimated from creatinine concentration, for cows grazing in the three pasture treatments showed that cows grazing both plantain and plantain–clover mix pastures produced on average 22 and 24% more urine (*p* ≤ 0.0001) in the 2017/2018 and 2018/2019 seasons, respectively (Figure 1). 

**Figure 2:**
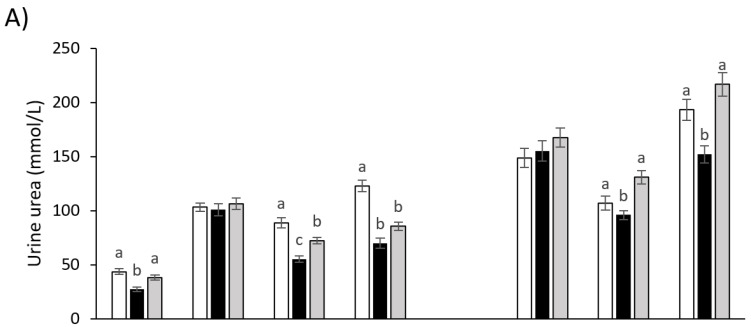

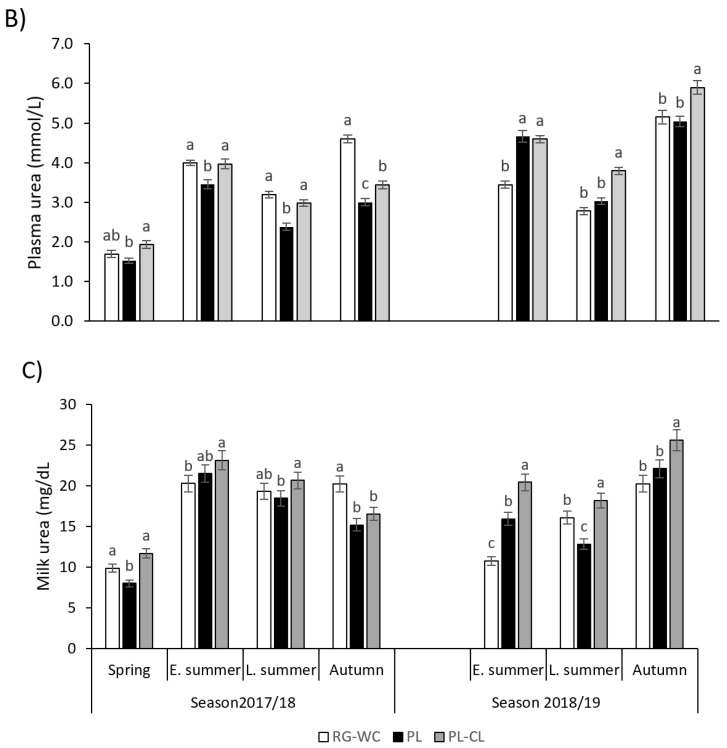
Urea concentration in (**A**) urine, (**B**) plasma and (**C**) milk of cows grazing plantain (black), plantain–clover (grey), and ryegrass–white clover (white) pastures in spring, summer (early and late), and autumn during the 2017/2018 (left) and 2018/2019 (right) lactating seasons. a,b,c letters indicate values in each season that are significantly different (*p* < 0.05).

The urine urea concentration for cows grazing plantain pastures was lower compared to those grazing ryegrass-wc pastures, but in early summer, in both 2017/2018 and 2018/2019 lactation seasons (Figure 2). The concentration of urea in the urine of cows grazing plantain-clover mix pastures was similar when compared to those grazing ryegrass-wc pasture, except in late summer and autumn of the 2017/2018 lactation season. Cows grazing plantain-clover mix pastures had a lower urine urea concentration than cows grazing ryegrass-wc pastures in late summer and autumn 2018. The concentration of urea in the plasma of cows grazing plantain pastures was lower in comparison to cows grazing ryegrass-wc pastures in summer (early and late) and autumn of the 2017/2018 season. During the 2017/2018 season, the plasma urea concentration for cows grazing plantain-clover mix pastures and ryegrass-wc pastures was similar, except in autumn 2018. In autumn 2018, cows grazing plantain-clover mix pastures had a lower plasma urea concentration than those grazing ryegrass-wc pastures (Figure 2). During the 2017/2018 lactation season, the milk urea concentration for cows grazing plantain pastures was lower in spring 2017 and autumn 2018 when compared to ryegrass-wc pastures, while in early and late summer cows grazing plantain and ryegrass-wc pastures had similar milk urea concentration. In the 2018/2019 lactation season, the milk urea concentration for cows grazing plantain pastures was higher in early summer 2018, lower in late summer 2019, and similar in autumn 2019 when compared to ryegrass-wc pastures. 

## 4. Discussion

This research on plantain-based pastures as an alternative pasture option for dairy cows to mitigate NO_3_^−^ leaching losses from grazed pastures, via lower UN loss from lactating cows, showed plantain reduced the UNc of lactating cows without a negative impact on MS production, when compared to ryegrass–wc pastures, over two lactation seasons. These results were in agreement with previous more short-term research [7,13,19,20,21], but showed the effects of plantain varied with season and changes in botanical composition of the pasture. Overall, the greatest reduction in N excretion by dairy cows was in autumn and when plantain was 30% or more of the pasture.

The N excreted in the urine of cows is widely known as the major source of N-NO_3_^−^ pollution into waterways from pastoral systems [3]. Modelling on the effect of UNc on N leaching from dairy farms indicated that reducing the UNc of cows by 23% via greater urine volumes per cow could lead to reductions in N leaching of up to 19% from farms [6]. Our research showed a decline of 35–40% in the UNc of cows grazing plantain pasture during late summer and autumn when compared to the urine of cows grazing ryegrass–wc pasture. The reduction in the UNc of urine patches deposited onto the soil when plantain was incorporated in the diet of cows implied a positive plant trait of plantain to mitigate the risk of nitrate leaching from cow’s urine [3,22,23]. This reduction in the UNc of cows, particularly during the critical period (late summer to autumn) for the accumulation of NO_3_^−^ in the soil when it is most susceptible to N leaching [5], reinforced the potential for plantain to mitigate N leaching from pastoral systems.

The decline in the UNc of cows grazing plantain pastures could be a consequence of dietary factors that increase urine production or that reduce the total UN excretion from cows [9,11]. In this research, despite the difference in the UNc, we found that plantain and plantain–clover mix pastures increased the urine production from lactating cows across both years of evaluation. The lower DM content and higher mineral concentration in plantain have been indicated to increase urine volume, as a consequence, in part, of a higher water intake from the herbage, reducing in this way the UNc of cows via a dilution effect [6,11]. Recent research, using urine sensors to measure urine volume from cows, showed that cows grazing plantain pastures produced 60% more urine volume daily in comparison with those cows grazing ryegrass–wc pastures [9]. Research on plantain has reported greater urine volume when more than 30% of the diet of the cows was plantain [13]. Greater urine volume from cows has been shown to increase the urination frequency of animals rather than the volume at each urination event [9,13,23]. The plantain trait for lower UNc via greater urine volume will potentially reduce the N leaching losses from pastoral soils under a similar total UN excretion by producing more urine patches with a lower N loading rate [12,23,24]. This finding suggested that the effect of plantain in increasing urine volume via higher urination frequency of cows, resulted in a greater spread of urine-N at lower N loading rates at each urination event, reducing the risk of N leaching from urine patches.

This research also showed the potential of plantain to reduce the amount of the dietary N excreted in the urine of lactating cows at similar or higher N intakes by changing the N partitioning in comparison to ryegrass–wc pastures. Nitrogen intake is the biggest factor influencing the amount of UN excreted by lactating cows, with around 80% of the daily N intake excreted [25]. This high proportion of N intake excreted by cows is due to an inefficient capture of rumen N as microbial protein and the metabolic cost of synthesizing and excreting urea to remove the accumulation of ammonia in the rumen. While reductions in N intake have a direct impact on lowering the UN excretion from lactating cows, improving the NUE can also have a positive impact by lowering N losses to the environment from dairy cows [25]. Previous studies have identified that higher WSC concentration in the diet of cows reduced the dietary N partitioned to urine and total UN excretion from lactating cows with more N being secreted in the milk [26,27]. Increasing the WSC concentration reduced ammonia (NH_3_) production in the rumen by increasing the synthesis of microbial protein in the rumen, leading to greater protein N absorption in the intestine and more protein secreted in the milk [28]. In our study, offering cows diets based on plantain or plantain–clover mix pastures did not exhibit clear benefits in regard to improving the NUE relative to ryegrass–wc pastures across both lactation seasons evaluated, and was in line with higher N intakes [25]. In this research, despite the higher N intake of cows, and that more N was in surplus (N intake minus N in milk), cows grazing plantain–clover mix pastures during the 2017/2018 lactation season were able to partition less dietary N to urine when compared to cows grazing plantain pastures. This plantain effect on the N partitioning from the mixed sward indicated a positive outcome for the environment by reducing the N excretion of the dietary N in surplus away from urine; however, this was not repeated in the 2018/2019 lactation season. The likely reason for this difference is the lower proportion of plantain in the cows’ diet in the 2018/2019 season (Table 2).

The presence of bioactive compounds (e.g., aucubin) in plantain has created interest in understanding whether their bioactivity could moderate N losses from dairy cows [29]. There is research which reported aucubin reduced NH_3_ production during ruminal fermentation, implying a lower amount of urea and total UN excreted by cows [14]. Milk urea concentrations are usually linked to NH_3_ production and are used as an indicator of the use of dietary N by rumen microflora, as this correlates well with urea and UN excretion [30]. The milk urea concentration observed for cows grazing plantain pastures suggested lower losses of N and urea in the urine compared to ryegrass–wc pastures, only during the 2017/2018 lactation season. In this research, from spring to late summer, cows grazing in the plantain pastures showed a similar total UN excretion as cows in the plantain–clover mix, despite the higher milk urea concentration and lower NUE. This effect on UN excretion could be in part due to aucubin influencing the NH_3_ and urea production during the ruminal fermentation and later digestion in the intestine. For example, the antimicrobial effect of aucubin on bacteria has been attributed to the aucubin aglycone (aucubigen) which binds to free amino acids (aa) making them unavailable [31,32]. The escape from the rumen of this aucubigen–aa union could also be moving the digestible N fractions to the intestine shifting the N partitioning from urine to faeces. Although the modes of action by which plantain reduced the UN excretion are not fully understood, they could include the influence of both aucubin and WSC in plantain changing the N partitioning.

During the second lactation season, an insufficient amount of plantain in the diet of cows was probably the reason for the lack of effect on UN excretion and N partitioning. Grazing studies have reported lower Une when cows obtained more than 40% plantain in their diet [7,21]. Recently, a metabolism stall study evaluating ryegrass–wc diets with increasing proportion of PL in the diet of cows showed that 30% of plantain is required in the diet of cows to produce reductions in the total UN excretion from lactating cows [13]. In this research, the average of PL in the diet of cows grazing plantain and plantain–clover mix pastures were 53% and 24% during the 2017/2018 and 2018/2019 lactation seasons, respectively. The lower % of plantain during the second year of evaluation supported the need to maintain an adequate amount of plantain in the diet of cows to modify the excretion of the dietary N surplus from lactating cows. A future challenge for the development of dairy systems based on plantain pastures will be ensuring sufficient plantain is available for the cows to exhibit the effect of plantain on N excretion. The benefit of plantain in reducing N leaching from dairy pastoral systems resulted from a greater number of urine patches with lower urinary N loading rates, combined with lower UN excretion. Despite these benefits of plantain, for the adoption of plantain by farmers, research needs to determine the direct effect on N leaching at farm level to assist in the adoption of plantain in New Zealand pastoral dairy systems.

## 5. Conclusions

Plantain pastures can be included in cows’ diets to create lower urinary N excretion without a loss in milk production and so provide a pasture option able to reduce the NO_3_^−^ leaching from NZ pastoral dairy systems. The relative ability of plantain to reduce N losses from lactating cows was associated with maintaining >30% of plantain in the sward and resulted from the combined effects of changing the N partitioning in the cow and increasing the volume of the cows’ urine.

## Figures and Tables

**Table 1:** Nutritive characteristic of plantain (PL), plantain–clover mix (PL-CL), and ryegrass–white clover (RGWC) pastures grazed by lactating dairy cows over two years.

Nutrients	2017/2018			SEM	*p*-Value	2018/2019			SEM	*p*-Value
	RGWC	PL	PL-CL			RGWC	PL	PL-CL		
Metabolizable Energy (MJ/kg DM)										
Spring	10.7	10.8	10.6	0.148	0.57	10.94 ^a^	9.74 ^b^	9.84 ^b^	0.172	0.0002
E. summer	10.9 ^a^	10.3 ^b^	10.3 ^b^	0.083	0.003	10.00 ^a^	9.00 ^b^	10.22 ^a^	0.149	0.0009
L. summer	9.8	9.6	9.9	0.133	0.38	8.64 ^b^	9.78 ^a^	9.94 ^a^	0.288	0.02
Autumn	9.4	9.6	9.4	0.280	0.82	10.62	10.94	10.48	0.161	0.24
Crude Protein (%DM)										
Spring	20.16 ^b^	16.02 ^c^	22.28 ^a^	0.606	0.003	22.06	20.04	22.26	0.906	0.16
E. summer	18.00 ^b^	16.92 ^b^	19.44 ^a^	0.608	0.03	19.20 ^b^	18.38 ^b^	24.18 ^a^	0.776	0.0005
L. summer	15.82 ^b^	14.96 ^b^	17.82 ^a^	0.446	0.0006	14.66	16.12	17.40	0.872	0.14
Autumn	19.24	17.98	20.16	1.445	0.46	21.72 ^b^	23.68 ^ab^	24.66 ^a^	0.749	0.04
Neutral Detergent Fibre (%DM)										
Spring	45.38 ^a^	28.04 ^b^	27.62 ^b^	1.099	<0.0001	45.76 ^a^	33.36 ^c^	37.80 ^b^	1.125	0.0001
E. summer	46.62 ^a^	34.30 ^b^	31.06 ^c^	0.887	<0.0001	46.46 ^a^	41.30 ^b^	44.70 ^a^	0.678	0.0001
L. summer	47.62 ^a^	37.26 ^b^	34.94 ^b^	1.086	<0.0001	50.28 ^a^	38.70 ^b^	37.56 ^b^	1.564	0.0001
Autumn	47.84 ^a^	34.86 ^b^	35.00 ^b^	2.351	0.007	46.52 ^a^	30.88 ^c^	36.64 ^b^	0.954	<0.0001
Non-Structural Carbohydrates (%DM)										
Spring	20.68 ^c^	44.02 ^a^	36.10 ^b^	1.369	<0.0001	18.18 ^b^	30.42 ^a^	25.12 ^a^	2.399	0.02
E. summer	23.98 ^b^	35.36 ^a^	37.68 ^a^	0.930	<0.0001	20.78 ^b^	25.30 ^a^	17.36 ^c^	0.948	0.0004
L. summer	23.78 ^b^	34.46 ^a^	35.16 ^a^	0.923	<0.0001	22.20 ^b^	30.84 ^a^	32.08 ^a^	0.607	<0.0001
Autumn	19.14 ^b^	30.40 ^a^	29.08 ^a^	2.138	0.01	17.78 ^c^	27.58 ^a^	24.10 ^b^	1.003	<0.0001
Water Soluble Carbohydrates (%DM)										
Spring	6.82 ^c^	13.60 ^a^	11.28 ^b^	0.400	<0.0001	6.86	8.60	7.86	1.024	0.52
E. summer	13.00	12.34	11.60	0.406	0.09	6.86 ^a^	1.98 ^c^	4.54 ^b^	0.487	0.0001
L. summer	8.48 ^b^	10.14 ^a^	10.80 ^a^	0.543	0.03	6.58 ^b^	8.20 ^a^	8.70 ^a^	0.233	0.0002
Autumn	5.62	6.80	6.42	0.538	0.34	6.06	6.00	6.58	0.424	0.61

^a,b,c^ superscripts indicate values in each season that are significantly different (*p* < 0.05).

**Table 2:** Total herbage harvested by lactating cows during the 2017/2018 and 2018/2019 lactation seasons and milk solids (MS) production, proportion of plantain in the total diet, and aucubin concentration in plantain (PL), plantain–clover (PL-CL), and ryegrass-white clover (RGWC) pastures in spring, summer (early and late), and autumn.

	2017/2018					2018/2019				
	RGWC	PL	PL-CL	SEM	*p*-Value	RGWC	PL	PL-CL	SEM	*p*-Value
Herbage Harvested (t DM/ha)	10.9 ^b^	10.1 ^b^	12.7 ^a^	0.27	0.04	10.3 ^a^	8.9 ^b^	10.7 ^a^	0.69	0.01
Milk Solids (kg MS/d)										
Spring	1.50	1.43	1.60	0.004	0.33	2.44	2.32	2.44	0.131	0.78
E. summer	1.59	1.33	1.17	0.154	0.17	1.99	2.05	1.97	0.069	0.67
L. summer	1.35	1.40	1.45	0.057	0.75	1.34	1.44	1.46	0.057	0.30
Autumn	1.27	1.16	1.19	0.051	0.26	1.01	1.10	1.06	0.118	0.71
Plantain in the diet (%)										
Spring	-	61	59	3.2	0.31	-	33	35	3.7	0.93
E. summer	-	50	48	6.3	0.78	-	27	10	4.9	0.06
L. summer	-	59 ^a^	35 ^b^	5.4	0.002	-	30	16	7.3	0.10
Autumn	-	46	40	3.7	0.21	-	22	10	4.3	0.12
Aucubin (g/kg DM)										
Spring	-	6.5	6.2	0.36	0.32	-	4.2	2.7	0.94	0.20
E. summer	-	6.5	5.1	0.44	0.06	-	1.4	0.4	0.44	0.28
L. summer	-	7.0 ^a^	4.1 ^b^	0.36	0.006	-	6.8 ^a^	2.7 ^b^	0.21	0.001
Autumn	-	2.8	2.1	0.94	0.63	-	6.1	3.3	0.75	0.06

^a,b^ superscripts indicate values in each season that are significantly different (*p* < 0.05).

**Table 3:** Nitrogen (N) intake, milk N yield, N use efficiency (NUE), and urine N excretion and N partitioned to urine by lactating cows grazing plantain (PL), plantain–clover (PL-CL), and ryegrass–white clover (RGWC) pastures in spring, summer (early and late), and autumn during the 2017/2018 and 2018/2019 lactation seasons.

	2017/2018					2018/2019				
	RGWC	PL	PL-CL	SEM	*p*-Value	RGWC	PL	PL-CL	SEM	*p*-Value
Nitrogen Intake (g N/d)										
Spring	659	400	486	58.9	0.07	474	380	480	55.9	0.19
E. summer	439	466	633	60.8	0.11	434 ^b^	367 ^b^	662 ^a^	54.8	0.001
L. summer	483 ^b^	669 ^a^	800 ^a^	64.9	0.003	388	449	403	52.7	0.72
Autumn	366 ^b^	340 ^b^	528 ^a^	41.9	0.009	739	719	940	157.1	0.57
Milk N (kg N/d)										
Spring	135.5 ^a^	121.5 ^b^	134.6 ^a^	3.7	0.01	187.2	181.1	187.2	7.1	0.79
E. summer	125.3	122.4	114.5	4.8	0.27	134.9	143.0	135.5	4.5	0.36
L. summer	95.6 ^b^	100.9 ^b^	107.8 ^a^	3.1	0.007	89.2	98.4	100.1	3.4	0.06
Autumn	92.6	86.5	92.6	3.3	0.29	75.6	87.6	84.5	8.6	0.62
NUE										
Spring	0.23 ^b^	0.31 ^a^	0.27 ^a^	0.031	0.04	0.47	0.48	0.41	0.069	0.55
E. summer	0.30 ^a^	0.27 ^a^	0.19 ^b^	0.027	0.03	0.35	0.32	0.20	0.049	0.07
L. summer	0.20 ^a^	0.15 ^b^	0.13 ^b^	0.016	0.01	0.24	0.22	0.28	0.035	0.55
Autumn	0.26 ^a^	0.27 ^a^	0.18 ^b^	0.024	0.008	0.16	0.17	0.11	0.047	0.59
Urine N (g N/day)										
Spring	124.9	109.7	123.8	7.45	0.27	-	-	-	-	-
E. summer	144.0	142.0	163.0	5.57	0.18	137.7 ^b^	179.8 ^a^	198.6 ^a^	8.79	0.003
L. summer	211.3 ^a^	154.3 ^b^	162.8 ^b^	7.38	0.002	108.4 ^b^	141.0 ^a^	151.3 ^a^	4.04	<0.0001
Autumn	142.3 ^a^	97.5 ^c^	131.9 ^b^	5.91	0.006	211.0 ^b^	210.9 ^b^	273.4 ^a^	11.33	0.01
N Partitioned to Urine										
Spring	0.21 ^b^	0.33 ^a^	0.30 ^b^	0.015	<0.0001	-	-	-	-	-
E. summer	0.49 ^a^	0.44 ^b^	0.33 ^c^	0.021	<0.0001	0.23 ^b^	0.37 ^a^	0.39 ^a^	0.020	<0.0001
L. summer	0.55 ^a^	0.26 ^b^	0.20 ^c^	0.019	<0.0001	0.27 ^c^	0.31 ^b^	0.37 ^a^	0.012	<0.0001
Autumn	0.59 ^a^	0.44 ^b^	0.32 ^c^	0.023	<0.0001	0.24 ^c^	0.36 ^a^	0.30 ^b^	0.018	0.02

^a,b,c^ superscripts indicate values in each season that are significantly different (*p* < 0.05).

## Data Availability

Data are available from the corresponding author on request.
